# Notes and News

**Published:** 1901-12-21

**Authors:** 


### Notes and News.

For the first time for upwards of 130 years Havanna is reported free of yellow fever. This fact is placed to the credit of the anti-mosquito crusade.

There are several cases of beri-beri in Liverpool. The patients are Lascars landed from a steamer from Rangoon. The Lascars were removed to the Southern Hospital. Two have died.

Many people will be glad to learn that Mons. Henri Dunant, the founder of the Red Cross organisations, has been awarded 75,000 kroner by the Alfred Nobel Peace Prize Committee. Not very long ago Mons. Dunant was in great poverty.

The four other Nobel prizes have been awarded at Stockholm to Dr. Emil Adolf Behring, of Hallé, for medicine; to Professor Hendrikus van Hoff, of Berlin, for chemistry; to Professor W. K. Rontgen, of Munich, for physics; and to Mons. Sully-Prudhomme, for literature.

The proposal of Dr. Wyman, Supervising Surgeon-General of the Marine Hospital Service of the United States, to establish an international sanitary commission, is an excellent one, if its labours can be carried out with sufficient discretion. The vexed question of quarantine, for instance, might be treated so as to avoid much soreness by a conference in which the interests of all might receive careful consideration, but whether the authorities of any suspect seaport will welcome the unsolicited recommendations of American deputies, even if associated with members of the nationality in which the seaport is situated, is another matter.

Mr. George Leuthal Cheatle, C.B., F.R.C.S., has been appointed surgeon to the Italian Hospital.

The course of the small-pox epidemic fluctuates from day to day, but there is practically no decided diminution, whilst the affected areas continue to spread. A case has appeared at Ipswich which originated, apparently, in London.

Mr. G. Mellin is placing at the disposal of the Prince of Wales's Hospital Fund a quantity of Mellin's food to the value of £10,000 for the use of the sick poor in the London hospitals. The distribution is to be spread over four years.

At the meeting of the Metropolitan Asylums Board, held last Saturday, it was resolved that it be a condition of service at the managers' imbecile asylums that all new officials and servants be vaccinated or re-vaccinated if the respective medical superintendents should so order.

The Prince of Wales has consented to become President of the National Association for the Prevention of Consumption. The association was inaugurated in 1898, the King being at that time President, and taking a very active interest in its proceedings. The chairman is Sir William Broadbent.

The value of the new arrangements made to educate crippled children of the London School Board is being recognised by the London County Council, who will award two scholarships to pupils of the Board, at the age of 16, to enable them to procure a suitable training in some occupation in which they can earn a living.

The Metropolitan Asylums Board have decided to increase their accommodation for small-pox cases from 1,740 to 2,500. The Holborn Borough Council asked the Board to arrange a conference of metropolitan sanitary authorities to discuss the subject of vaccination as a preventive measure, but the Board decided to refuse.

At a special Court of the Governors of the North London Hospital for Consumption held last week it was decided that the hospital should be known in future as "The Mount Vernon Hospital for Consumption." It was stated that the change of name was desirable in order to avoid clashing with the University College Hospital, which is also known as the North London Hospital.

An amusing discussion as to the difference between corpses and mummies took place during the recent case in the Law Courts to recover damages for injury of a mummy. During the trial the Judge alluded to the mummy as a corpse. The barrister representing the owner denied that a mummy was a corpse. To the riddle propounded by the Judge, "When does a corpse cease to be a corpse?" the barrister replied: "When it was once a mummy—after which it always remained a mummy." But as a matter of fact, the sea voyage appeared to have converted the mummy into something so nearly a corpse again that its burial was demanded by the sanitary authorities.

